# Cognitive Inflexibility Predicts Negative Symptoms Severity in Patients with First-Episode Psychosis: A 1-Year Follow-Up Study

**DOI:** 10.3390/brainsci14020162

**Published:** 2024-02-05

**Authors:** Leonidas Mantonakis, Pentagiotissa Stefanatou, Antonis Tsionis, George Konstantakopoulos, Lida-Alkisti Xenaki, Angeliki-Aikaterini Ntigrintaki, Irene Ralli, Stefanos Dimitrakopoulos, Konstantinos Kollias, Nikos C. Stefanis

**Affiliations:** 1 First Department of Psychiatry, Eginition Hospital, School of Medicine, National and Kapodistrian University of Athens, 11528 Athens, Greece; lmantonakis@gmail.com (L.M.); ant.tsionis@gmail.com (A.T.); gekonst@med.uoa.gr (G.K.); kalamaraki@yahoo.com (L.-A.X.); aggeliki.aikaterini@gmail.com (A.-A.N.); ireneralli@gmail.com (I.R.); stefandimi13@gmail.com (S.D.); kollias@med.uoa.gr (K.K.); nistefan@med.uoa.gr (N.C.S.); 2 Research Department of Clinical, Education and Health Psychology, University College London, London WC1E 7HB, UK; 3 Psychiatric Clinic, 414 Military Hospital of Athens, 15236 Palea Penteli, Greece

**Keywords:** first episode psychosis, FEP, cognitive deficits, negative symptoms, executive functions, cognitive flexibility, Wisconsin card sorting test, longitudinal study, prediction, PANSS

## Abstract

Negative symptoms and cognitive deficits play a major role in psychosis and significantly influence the functional outcomes of patients, particularly those with a first episode of psychosis (FEP). However, limited research has explored the predictive capacity of cognitive deficits during FEP for subsequent negative symptomatology. Drawing from the Athens FEP research study, we conducted a retrospective longitudinal study in 80 individuals with FEP. All patients were drug naive at admission. Cognitive tests were administered at 1-month and 1-year post-admission, while negative symptomatology was assessed at the same time points using PANSS by trained raters. We considered confounding factors such as age, gender, duration of untreated psychosis (DUP), treatment received, premorbid social adjustment, and premorbid IQ. Univariate regression analysis identified cognitive domains that correlated with negative symptomatology. These, along with the confounders, were incorporated into a multiple regression, with the 1-year PANSS negative scale serving as the dependent variable. Employing the backward elimination technique, we found a statistically significant inverse relationship between the categories completed in the Wisconsin card sorting test (WCST) and the 1-year PANNS negative scale (*p* = 0.01), beyond the associations with DUP and the 1-month PANSS negative scale. Our results suggest that cognitive flexibility, a key component of executive functions, predicts negative symptom severity one year after FEP.

## 1. Introduction

Psychotic disorders represent a complex and enduring psychopathology, exerting profound effects on diverse aspects of mental functioning such as thinking, perception, volition, emotion, and cognition. The repercussions of this intricate condition extend to the disruption of an individual’s connections with both the self and the external world [1]. Within the clinical realm, psychosis presents itself with a spectrum of phenotypes and an array of symptoms. Key manifestations encompass delusions, hallucinations, disordered thinking, grossly disorganized or abnormal motor behavior (including catatonia), as well as negative symptoms [2,3]. The diagnostic landscape of psychotic disorders is extensive, with numerous conditions falling under the umbrella of psychosis. As outlined in the DSM-5, recognized diagnoses include schizophrenia, brief psychotic disorder, delusional disorder, schizoaffective disorder, schizophreniform disorder, and schizotypal (personality) disorder. Additionally, psychosis can be induced by substances/medications or arise due to another medical condition. Moreover, it is noteworthy that psychosis is not confined solely to primary psychotic disorders; it can also manifest with certain mood disorders. Examples of such comorbidity encompass bipolar disorder and major depression with psychotic features [4].

Negative symptoms constitute a fundamental feature of psychotic disorders [3], associated with poor functional outcomes [5], inadequate response to treatment [6], and difficulties in social, professional, and personal aspects of patients’ lives, leading to significant impairment [7]. Many studies have demonstrated that levels of negative symptoms in FEP individuals have been associated with both their current and long-term functional impairments. Specifically, at the initial evaluation, FEP patients with more pronounced negative symptoms tend to experience greater difficulties in areas such as work, social relationships, and independent living, both at the time and in the future [5,8,9,10,11]. However, despite their debilitating impact on patients’ psychosocial functioning, negative symptoms are often under-recognized, undertreated, and widely considered an unmet therapeutic need [12].

Cognitive dysfunction is also a core feature of psychosis that strongly correlates with poorer clinical and psychosocial outcomes, as well as a decrease in patients’ quality of life [13]. These deficits often precede the onset of the illness, endure during the transition from prodromal stages to full-blown disorder [14], and persist even after the remission or resolution of positive psychotic symptoms [15]. More specifically, meta-analytic evidence has unequivocally indicated that cognitive deficits in FEP patients are a central predictor of their current and future psychosocial functioning [16]. Importantly, cognitive impairments significantly and negatively affect functional recovery, even after controlling for positive symptoms, negative symptoms, and DUP [17]. Patients experiencing FEP have been found to exhibit cognitive deficits similar to those observed in chronic patients [18]. Notably, a meta-analysis of studies on FEP patients who had not received medication found significant cognitive impairments across various domains compared to healthy controls, with the most pronounced deficits in verbal memory, processing speed, and working memory [19].

A recent meta-analysis [20] explored the relationship between the neurocognitive domains defined by the MATRICS consensus and negative symptoms in schizophrenia. The results demonstrated modest yet statistically significant negative correlations across all domains, with a stronger connection to the expressive aspects of negative symptoms.

In their systematic review, Melillo et al. [21] identified in FEP associations between negative symptoms and an extensive array of cognitive impairments, encompassing deficits in executive functions, theory of mind, working memory, verbal fluency, and importantly, processing speed. Worse cognitive performance was noted in subjects with persistent symptoms. Furthermore, the review identified inconsistent findings in the relationship between negative symptoms and various cognitive areas, notably in processing speed, verbal fluency, attention/vigilance, working memory, and overall social cognition. They report that several studies have identified no specific cognitive domain associated with negative symptoms, while others have noted a negative correlation with the aggregate cognitive scores.

Another systematic review [22], spanning studies from 2009 to 2022, reveals a slight escalation in cognitive impairments from FEP patients to the chronic stage of the illness, underscoring the presence of broad cognitive impairments already at FEP.

The potential interplay between negative symptoms and cognitive deficits is of clinical importance because both these factors occur early in the illness and have a significant impact on patients’ functioning [23].

In conclusion, recent research reveals that patients with FEP exhibit cognitive deficits similar to chronic stages, particularly in verbal memory, processing speed, and working memory. Meta-analyses and systematic reviews have identified modest but significant negative correlations between various cognitive impairments, including executive functions and working memory, and negative symptoms in schizophrenia, with notable inconsistencies in specific cognitive domains associated with these symptoms [11].

Only a few longitudinal studies thus far have investigated the relationship between cognitive deficits and negative symptoms in FEP patients. A study that examined the predictive power of cognitive deficits and insight on outcomes using a battery consisting of the Wechsler Adult Intelligence Scale (WAIS) III, Trail Making Test (TMT) A and B, the Cambridge Neuropsychological Test Automated Battery, and a verbal semantic fluency test found that the performance in TMT B on admission predicted the severity of negative symptoms after one year. Νo association was found between TMT-A and negative symptoms [24]. Another study which included measures from WAIS, the WMS, the TMT, verbal fluency, the tapping task, and the continuous performance test identical pairs found cognitive functions to be correlated inversely with negative symptoms but not with positive or affective symptoms one year after admission [25]. A third study, revealed that improvements over time in general cognitive function, working memory, and verbal learning were mediated by improvements in positive and negative symptomatology [26]. In a different study [27], FEP participants were categorized into different negative symptom groups regarding their negative symptoms trajectories during one year of follow-up: sustained negative symptoms (SNS), mild (MNS), transitory symptoms (TNS), and no negative symptoms (NNS). The following measures of cognition were included: the abbreviated WAIS, California Verbal Learning Test, the WMS, and the D-KEFS test battery. The SNS group had a significantly poorer performance in executive functions and processing speed compared to the NNS group, and poorer verbal learning and memory compared to the MNS and TNS group. The NNS group’s cognitive performance was comparable to that of healthy controls [27]. On the other hand, another prospective study found that FEP patients that had persistent negative symptoms one year after admission did not show significant differences on neuropsychological indices at baseline, compared to those without persistent negative symptoms [23]. Finally, a prospective study of FEP patients, which encompassed WAIS, WMS, the Letter-Number Span Test, the monotone counting test, the modified WCST, the TMT A and B, category verbal fluency, the Hayline Sentence Completion test, and Modified Six Elements test, found that baseline amotivation levels and the number of perseverative errors committed in modified WCST and TMT part B performance, independently predicted amotivation at 6-month follow-up [28].

Summarizing, recent longitudinal studies reveal a complex relationship between cognitive deficits and negative symptoms in FEP, but findings vary. Some studies show specific cognitive tests to predict negative symptom severity [15,19] and poorer cognitive performance [18] in executive functions among patients with sustained negative symptoms. Yet, other research [14] presents contrasting results, including no significant cognitive differences, at baseline, in FEP patients with different negative symptom trajectories.

Certain factors may be confounders in the relationship between cognitive impairments and the course of negative symptomatology in the early stages of psychosis. The duration of untreated psychosis (DUP) is significantly associated with negative symptoms. A shorter DUP is linked to fewer negative symptoms, both in the short and long term, particularly if DUP does not exceed 9 months [29]. In addition, poor premorbid social functioning [30], as well as low premorbid IQ [31], have been correlated with persistent negative symptoms and a poorer treatment response in patients with FEP.

Prior studies have established a link between cognitive deficits and negative symptoms, yet some findings remain contradictory or inconsistent [12,14,18]. Studies have often focused on specific cognitive functions, employing select neuropsychological tests or subtests [16], and occasionally overlooking others. In a study where cognitive deficits predicted amotivation, dimensions of negative symptoms were assessed and not negative symptoms as a whole [19]. Furthermore, the influence of confounders such as premorbid functioning is often not considered. To address these gaps, we designed a longitudinal study examining the predictive capacity of various cognitive deficits for negative symptoms in FEP patients one-year post-admission. Our study encompasses a broad spectrum of cognitive deficits, addresses negative symptoms as a whole and accounts for significant potential confounders, including the DUP, premorbid IQ, and premorbid social functioning.

The aim of this study, spanning the initial treatment period, was to determine if cognitive impairments in drug-naive FEP patients can predict the severity of negative symptoms one year later. Our objectives were: (1) to identify the specific cognitive deficits with the greatest predictive value for future negative symptomatology in the early stages of psychosis; (2) to examine the influence of major demographic factors and premorbid social adjustment, as well as DUP, treatment received, and premorbid IQ on the relationship between cognitive deficits and negative symptoms.

## 2. Materials and Methods

This study employs a retrospective longitudinal design, in order to utilize the data obtained from the “Athens First-Episode Psychosis Research Study” from 2015 to 2020. This design allows us to observe how variables have changed or influenced each other over a certain period.

### 2.1. Study Participants

The data of 80 patients were sourced from the “Athens First-Episode Psychosis Research Study” database. Up until recently, Eginition University Hospital has been the only provider in Athens offering a wide range of services for early and long-term psychosis intervention. In partnership with four other hospitals in Athens, all of which offered standard care, Eginition Hospital launched the Athens first-episode psychosis research study, a prospective longitudinal study conducted between 2015 and 2020. The strategies employed to establish the study cohort, encompassing participant selection, recruitment, assessment, and overall framework, have been discussed elsewhere [32]. The baseline for the FEP patients in the study was defined as their initial admission at the emergency departments of these hospitals.

The patient group at baseline consisted of individuals aged 16–45 experiencing their first episode of psychosis, diagnosed according to the ICD-10 classification. Exclusion criteria encompassed various factors such as organic causes of psychotic symptoms, low IQ (<70), developmental deficits, and prior exposure to antipsychotic drugs (≤2 weeks). The Research Ethics Committee of Eginition Hospital granted approval for the research under protocol number 644Υ46Ψ8Ν2-ΓΚΣ, 17 February 2015. All eligible subjects provided written informed consent.

Due to the inherent challenges associated with accurately assessing cognitive functions during the acute phase of the disease, the first evaluations included in this study were conducted one month after the admission of FEP patients (1st measurement), at a timepoint when all patients were under treatment. The 2nd measurement occurred at the 1-year follow-up. A multidisciplinary team of mental health researchers carried out the assessments. Clinical assessments were conducted by clinicians, while neuropsychological assessments were performed by qualified neuropsychologists.

### 2.2. Clinical Assessments

Τhe Positive and Negative Syndrome Scale (PANSS) was used for the assessment of symptoms. It consists of three subscales: the positive scale (PANSS positive), the negative scale (PANSS negative), and the general psychopathology scale (PANSS general psychopathology) [33,34]. Numerous models have emerged from factor analyses of the positive and negative syndrome scale (PANSS), with some incorporating cognitive dimensions. In our study, we employed the original three scales of the PANSS in our analysis. This enabled the incorporation of the negative scale, encompassing all negative symptoms comprehensively.

The Nottingham Onset Schedule: modified DUP version [35] was used for the calculation of DUP.

### 2.3. Premorbid Adjustment Assessments

We assessed premorbid adjustment using the Premorbid Adjustment Scale (PAS-GR). The PAS-GR evaluates individual functioning across four key developmental stages: childhood (up to age 11), early adolescence (ages 12–15), late adolescence (ages 16–18), and adulthood (age 19 and onwards). The PAS was administered by a trained psychiatrist based on interviews with patients and their family members, predominantly parents, ensuring that these family members had maintained a close relationship with the patient during their childhood and adolescence. During these interviews, the PAS rater chose scores that best matched the descriptive phrases provided by the interviewees. The scale facilitated the estimation of social adjustment through the evaluation of peer relationships, sociability/withdrawal, and socio-sexual interactions in each developmental period, with the note that socio-sexual ties are excluded in the childhood stage. Academic adjustment was gauged using the items related to scholastic performance and school adaptation at each developmental stage. Notably, the late adolescence and the adulthood subscale were excluded from our statistical analysis to eliminate potential confounders arising from symptoms manifesting in the prodromal phase [36,37,38].

### 2.4. Cognitive Assessments

General intellectual ability was assessed using the Greek version of the Wechsler Adult Intelligence Scale—fourth edition (WAIS-IV^GR^) [39,40]. This comprehensive neurocognitive battery comprises 10 basic subtests grouped into four cognitive indexes: verbal comprehension (VCI), perceptual reasoning (PRI), working memory (WMI), and processing speed (PSI). Raw scores were converted to age-corrected scaled scores using Greek norms for determining both the four cognitive indices and the full-scale IQ (FSIQ).

Premorbid IQ was assessed by utilizing the vocabulary subtest of WAIS-IV^GR^ in accordance with existing literature and international practices [41].

Executive functions were assessed through (a) the Wisconsin Card Sorting Test (WCST) employing the Greek norms [42,43]; this involved a detailed analysis of various performance metrics, including percent errors, percent perseverative responses, percent perseverative errors, percent conceptual level responses, and the number of Categories completed, and (b) the trail making test B (TMT-B) Greek version [44,45].

Attentional functions such as scanning, processing speed, visuomotor tracking, and co-ordination were evaluated using the Trail Making Test A (TMT-A) [44,46].

### 2.5. Treatment Assessment

To assess the impact of treatment on the evolution of negative symptoms at the one-year follow-up, we calculated according to established standards and utilized the chlorpromazine equivalent (CE) tailored to the treatment received by each participant at the one-year follow-up [47,48].

### 2.6. Statistical Analysis

While analyzing our dataset, we encountered instances of missing data. Specifically, PAS data were unavailable for seven subjects because they lacked family members available for interviews. Additionally, six subjects were unable to complete the WCST, and one could not undergo the WAIS-GR test, due to the unavailability of trained neuropsychologists at their respective contact hospitals during the data collection period. The Nottingham DUP scale was inapplicable for two subjects, and one subject was not available for the one-year follow-up. The dosage information for the treatment of one patient was unavailable. After removing records with missing data, our refined dataset included 63 patients. For these patients, we successfully recorded the specified measures at both the initial 1-month and the subsequent 1-year intervals.

We performed a univariate regression analysis to determine the variables that would be included in the multiple regression model. As independent variables, we considered, one at a time, all cognitive function measure scores at the 1-month time point, along with age, gender, education years, 1-month PANSS scores, PAS academic and social scores, premorbid IQ, and the treatment received in the 1-year follow-up in CE. The dependent variable was the 1-year PANSS negative score. To prevent the exclusion of variables that might exhibit increased correlation in a multiple regression model, we adopted a slightly lenient threshold of *p* < 0.2.

All variables that presented a significant association with the 1-year PANSS negative score in univariate regression analysis were included as independent variables in a multiple regression, with the 1-year PANSS negative score serving as the dependent variable.

To evaluate multicollinearity concerns we implemented the variance inflation factor (VIF). Variables with high VIFs, indicating problematic multicollinearity, were subsequently excluded by our multiple regression.

The fitness of this starting model was evaluated using various diagnostic tools, these encompassed assessments of goodness-of-fit statistics, including R-squared and adjusted R-squared values. Additionally, residual analysis, normality checks, and scrutiny of influential data points were conducted to validate assumptions.

To refine the model, a backward elimination approach was employed, utilizing a threshold of *p* < 0.5. To assess the model’s fitness at each step, we employed both clinical criteria and quantitative measures, including the Akaike information criterion (AIC) and the Bayesian information criterion (BIC).

All data were imported and processed in RStudio, utilizing the R programming language.

## 3. Results

### 3.1. Demographic and Clinical Characteristics

Our study includes a total of 63 patients, comprising 37 males and 26 females. The mean age of all the participants is 26.3 years, with an age range spanning from 16 to 46 years. The summary of the details of our sample can be found here (Table 1).

### 3.2. Univariate Regressions

The univariate analysis revealed correlations (*p* < 0.2) between the 1-year PANSS negative score and the following variables: DUP, CE, age, gender, premorbid IQ, 1-month positive PANSS, 1-month negative PANSS, 1-month general PANSS, social PAS, VCI, categories completed, percentage errors, perseverative errors, and percent conceptual level response (Table 2).

### 3.3. Multiple Regression Analysis

Due to high multicollinearity, verified through the variance inflation factor (VIF), both percentage errors and percent conceptual level response were excluded from the model.

After this exclusion, all remaining variables entered a starting multiple regression model as independent with the 1-year negative PANSS serving as the dependent variable.

The residual standard error of the starting model was 4.471 on 50 degrees of freedom, the multiple R-squared: 0.49 and the adjusted R-squared: 0.37 with an F-statistic: 4.069 on 12 and 50 DF, and the *p*-value was 0.0002068.

Additionally, residual analysis, normality checks, and examination of influential data points were performed to ensure the validity of assumptions. The Shapiro–Wilk test was conducted on the residuals of the initial multiple regression model, prior to the commencement of backward elimination. The results revealed a W statistic of 0.98465 with a corresponding *p*-value of 0.6207. This suggests that the assumption of normality for the residuals is met, indicating that the residuals follow a normal distribution.

The resulting model, outlined in Table 3, was achieved through successive elimination steps.

In the final model, the adjusted R-squared is approximately 0.38, the *p*-value associated with the model is remarkably low (7.379 × 10^−7^), the residual standard error is 4.448, based on 57 degrees of freedom, and the F-statistic is calculated at 13.62 with degrees of freedom of 3 and 59. The post hoc power analysis of the final model was 99%.

In our analysis, the 1-month PANSS negative score was strongly and positively correlated with the 1-year PANSS negative score (β = 0.344, *p* < 0.001.)

Moreover, a significant positive relationship between DUP and the 1-year PANSS negative score (β = 0.032, *p* < 0.001) was found.

Finally, the number of categories completed in the Wisconsin card sorting test was identified as a significant predictor (β = −0.812, *p* = 0.0104). This factor had the most considerable effect size among the predictors we examined, highlighting its critical influence on the 1-year PANSS negative score.

## 4. Discussion

This study aimed to investigate for the potential of cognitive deficits to predict subsequent negative symptom development in FEP patients. Utilizing a longitudinal research design and carefully accounting for confounding variables, our goal was to identify deficits in specific cognitive areas that can serve as predictors of negative symptoms one year after admission. We found that the WCST categories score, along with the 1-month PANSS negative score and DUP, significantly predicted the level of negative symptoms in FEP patients one year later. Among these predictors, WCST categories had the most pronounced effect on negative symptoms one year after admission.

Categories score is considered a significant measure for assessing abstract reasoning, concept formation, and cognitive flexibility (CF) [49], and the latter represents a key component of executive functions, also called set shifting or mental flexibility [50]. Successfully completing a category in WCST involves recognizing and shifting cognitive strategies in response to changing environmental stimuli [50]. A higher number of categories completed generally indicates better CF, adaptive problem-solving skills, effectively switching between mental processes, and the ability to display flexibility in the face of changing schedules of verbal reinforcement (i.e., feedback information from the examiner) [51]. It is worth noting that the WCST is one of the most widely employed measures of executive functioning in the context of schizophrenia research, while the categories score is amongst the scores most frequently analyzed in psychotic illnesses [52]. Individuals with schizophrenia have been found to succeed at significantly fewer categories than healthy controls [53]. Furthermore, research has shown that there is considerable cognitive heterogeneity among individuals with psychosis on the WCST, indicating the existence of distinct cognitive phenotypes within this group, ranging from relatively intact to severely impaired cognitive functioning [54].

The inverse relationship between the categories and the 1-year PANSS negative score in our study suggests that deficits in CF, specifically in the ability to shift cognitive strategies in response to environmental changes, contribute to the persistence and severity of negative symptoms.

Our results are aligned with those reported by Chang et al. [28]. In their study in FEP patients, they found that baseline deficiencies in switching and flexibility are predictors of amotivation in FEP at the 6-month and 1-year follow-up. In their research, these aspects were assessed through the count of perseverative errors, which is also considered a cognitive flexibility score [49] in a modified version of WCST (MWCST). Additionally, the categories score was a notable predictor at a 6-month follow-up in their study, though it ceased to be significant at the 1-year follow-up. It should be noted that Chang et al. examined predictors of a specific dimension of negative symptoms, i.e., amotivation, while the present study examined the negative syndrome as a whole. Moreover, the difference in results may be due to the different versions of WCST employed between studies. Specifically, MWCST is a simplified and less demanding version related to set shifting, as the examinee is alerted by the examiner when the rule of sorting is changed and is instructed to find another sorting rule. However, this modification makes the task easier, facilitating the achievement of more categories. Furthermore, given that there is not a consensus structure both for executive functions and WCST factor structure [49,50], Chang et al. included categories in a different component of executive functions, that of planning and strategy allocation instead of switching and cognitive flexibility.

Our findings are also in line with those of O’ Connor et al. [24] who found in a group of FEP patients that the severity of negative symptoms at 12-month follow-up was predicted by deficits in executive functions at baseline, as estimated by the TMT-B test while no other cognitive variable contributed to the prediction. However, WCST was not included in that study.

Carlsson et al. [55] found that cognitive deficits in FEP patients at admission were able to significantly predict the negative symptoms at a 1-year follow-up, whereas diagnosis, symptom severity, and DUP were not found to have predictive value in this respect. In their research, WAIS-R full-scale IQ was the only neurocognitive predictor examined. On the contrary, our study searched for predictors of negative symptom severity across a broad range of cognitive deficits. However, in our research, WAIS-IV indices or full-scale IQ did not predict negative symptoms after 1 year, and the WCST categories score emerged as the only significant cognitive predictor, suggesting that impairments in cognitive flexibility may have a greater contribution to the severity of negative symptoms than general intellectual ability. This discrepancy in findings may be attributed to methodological issues. More precisely, the study they conducted utilized an earlier edition of the WAIS (WAIS-R), whereas our research employs the most recent version of the assessment (WAIS-IV), which features newly developed subtests that were not included in previous versions and omits some older subtests. Additionally, the Carlsson et al. research divided patients into high and low negative symptom groups using a different scale (BPRS) and examined negative symptom severity as a categorical variable, while our study regarded negative symptom severity as a continuous variable.

González-Ortega et al. studied FEP patients [56] using a battery of tests, including the WAIS-III and WCST among others, and found that working memory, as measured by WAIS-III, was a predictor of negative symptoms at a 5-year follow-up in 26 FEP patients. They found no significant predictive role of WCST scores, but they did not include the categories score in their analysis. In contrast, our study found that working memory estimated by the WMI of WAIS-IV did not predict negative symptoms one year after FEP. Yet, in the González-Ortega et al. study working memory was estimated through the WAIS-III digit span and letter-number sequencing subtests. The latter is a measure of executive working memory [57], which is not included in the core subtests of WAIS-IV and as a result was not administered in our study participants. Thus, bearing in mind that letter-number sequencing and the WCST categories are both measures of CF, we could assume that our findings and those of the González-Ortega et al. study exhibit some points of convergence. Conversely, the results inconsistencies among studies may be related to illness-stage differences between the two distinct study groups. Specifically, our research aimed to determine the extent to which risk and protective factors could predict the development and progression of negative symptoms in drug-naive FEP patients one year after admission and, indirectly, the treatment responsiveness. Thus, our study centered on the initial stages of psychosis, avoiding the confounding factors of the established stage of the illness that the participants in the González-Ortega et al. study were at during the time of re-examination [58,59].

During a study performed by Üçok et al. [60] on FEP, patients were divided into groups based on the presence and persistence of negative symptoms. They differentiated patients with persistent negative symptoms (PNS) from those without (no-PNS), based on specific criteria across the two-year period. The study found that PNS had an earlier onset of disease, lower premorbid functioning, and more pronounced deficits in executive functions and attention. The tests included the Rey auditory verbal learning test, Stroop test, WCST, digit span test, continuous performance test, TMT, and the *n*-back test. The PNS group exhibited a poorer performance in forward digit span, number of correct responses, and completed categories in the WCST. Additionally, a negative correlation was found between DUP and correct responses in the WCST. These findings are in harmony with our findings about the importance of CF and its relationship with negative symptoms. On the other hand, working memory, attentional focus, and concentration as measured by the subtest of WAIS and TMT A and B did not serve as predictors of negative symptoms in our study. It is important to note that in the Üçok et al. study, the cognitive test battery was administered only to the last 53 participants of their sample; thus, it might not represent the entire sample adequately. This could limit the generalizability of the findings, as cognitive performance data were not available for all participants. Additionally, this study used binary logistic regression to identify independent variables contributing to PNS at one year. This analysis method focuses on determining factors that distinguish between two groups (PNS vs. no PNS) at a specific time point. While useful for identifying associations, it may not fully capture the complexity and evolution of symptom severity over time. In our study, premorbid functioning measured through PAS did not predict negative symptoms after one year.

To ensure the robustness of our results, our analysis included a broad range of other potential predictors, such as demographic and clinical characteristics, treatment received at 1-year follow-up, DUP, premorbid adjustment, and premorbid IQ. Moreover, by considering possible confounding factors, our study seeks to provide a more comprehensive and accurate perspective on the interplay between cognitive deficits and negative symptomatology.

The positive association of DUP with the 1-year PANSS negative score in our study is in agreement with the previous findings discussed below [61]. Advanced synthesis of existing data through a recent umbrella review and random-effects meta-analysis suggests a consistent pattern: longer DUP correlates with more pronounced negative symptoms both at initial presentation and during follow-up [62]. This pattern is corroborated by the findings of our study.

In a study by Molina Garcia et al. [31], FEP patients with an early onset and low premorbid IQ have been found to present persistent negative symptoms, lower functioning, and less recovery likelihood at two-year follow-up. In our study, premorbid IQ has no predictive value of negative symptoms at follow-up, indicating that the impairment in executive functions exerts a stronger impact on the progression of negative symptomatology and the divergence could also be attributed to the influence of early onset in prior findings.

In our study, the severity of negative symptoms at one month was found to be a predictive factor for their severity at the one-year follow-up. This is in agreement with previous findings, such as those by Chang et al. [28], discussed above and a prospective study focused on the course of negative symptoms in FEP patients by Mezquida et al. [63], where negative symptoms at baseline predicted negative symptoms at 1- and 2-year follow-up. This observation underscores the resistance of these symptoms to current treatment, their enduring nature over time, and their status as an important unmet therapeutic need. Moreover, this finding highlights the necessity for further research to develop effective treatments for the enduring negative symptoms [64].

A study by Mezquida et al. [63] investigated negative symptoms in first-episode schizophrenia over a two-year period. The study found a significant reduction in negative symptoms after one year, which remained stable into the second year. Notably, the study highlighted that poorer premorbid adjustment and higher baseline negative symptoms significantly predicted more severe negative symptoms at two years. However, it did not focus specifically on cognitive deficits and their predictive value for negative symptoms. In our study, cognitive deficits and specifically CF served as a predictor of negative symptoms after one year, whereas premorbid adjustment did not.

Starzer et al. [61] conducted a 20-year analysis of symptom trajectories in schizophrenia spectrum disorders post-FEP. The study identified various trajectories for both positive and negative symptoms, noting that nearly half of the patients experienced persistent negative symptoms. These continuous negative symptoms were associated with lower social and neurocognitive functioning, as indicated by reduced scores in brief assessment of cognition in schizophrenia (BACS) scores, reducing the likelihood of symptom remission. These findings align with our research on the significant interplay between cognition and negative symptoms, highlighting the importance of the association, which remains significant after 20 years. Furthermore, this study also highlights the predictive power of DUP on the stable trajectory of negative symptoms. In the study, lower global functioning and incomplete education years serve as predictors of persistent negative symptoms. In our study, CF is a stronger predictive factor of negative symptoms than education.

In our study, the treatment received at the 1-year follow-up did not show a significant association with the severity of negative symptoms at that specific time point. This lack of correlation may be attributed to the relatively stable course of negative symptoms [5], known for their limited responsiveness to standard treatment modalities [6]. Despite close supervision by therapeutic teams throughout the follow-up period, patients received standard care due to the non-operational status of early intervention in psychosis services [65]. Integrated treatment, identified as potentially beneficial in alleviating negative symptoms [66], was not available. The evaluation of treatment, measured in chlorpromazine equivalents, primarily reflects the administration of antipsychotic medication, known to have limited effectiveness against primary negative symptoms [12]. However, given the constraints of our sample size, caution is warranted in interpreting these findings. Future research within the Greek FEP population is crucial for a comprehensive assessment of negative symptom trajectories in response to different medication regimens. Additionally, investigating the impact of new early intervention services, compared to conventional approaches, on negative symptom trajectories is imperative. This targeted research will offer valuable insights into the effectiveness of emerging treatment modalities and guide the optimization of interventions for individuals experiencing first-episode psychosis.

Again, it is worth emphasizing that both cognitive deficits and negative symptoms are crucial determinants of future functioning for FEP patients. Remarkably, the simultaneous predictive relationships of negative symptoms and cognition with functionality has been a recurring finding in numerous studies across psychotic illness-stages, including clinical high-risk state for psychosis (CHR) [67,68], FEP [69,70], and chronic populations [71]. This has lent support to the hypothesis of some overlap between negative and cognitive symptoms and highlighted the intricate interplay among cognition, negative symptoms, and functioning, with mixed findings in prior reports. In FEP patients, meta-analytic findings have demonstrated the substantial relationship of cognition with future psychosocial functioning, after accounting for negative symptoms [17], whereas a separate report found that the inclusion of negative symptoms in the analysis eliminated the predictive power of cognition for 1-year outcomes, underscoring the intersection between negative symptoms and cognition [25]. Moreover, some reports in CHR individuals have shown a predictive relationship between cognitive variables and functioning through the partial mediation of negative symptoms [67,68]. Notably, our findings partially reflect those of studies in CHR individuals, which suggest that CHR converters to psychosis exhibit greater cognitive impairments than non-converters [72,73], resulting in poorer functional outcomes [74]. More specifically, the meta-analysis by Fusar-Poli et al. [75] revealed that individuals with a vulnerability to psychosis experience neurocognitive deficits qualitatively comparable to those of FEP and schizophrenia patients, namely in executive functions, verbal fluency, attention, visual and verbal memory, and working memory. Additionally, the severity of negative symptoms has been found to be a key predictor of transition to psychosis in CHR individuals, independently of cognitive impairments [76], and also a determinant of impaired global and social functioning [77,78].

Neuroimaging studies reveal overlapping brain structures in the neurobiological basis of CF and negative symptoms. CF is linked to various brain structures, including the dorsolateral prefrontal cortex (DLPFC), basal ganglia, thalamus, anterior cingulate cortex, inferior frontal junction (IFJ), inferior parietal lobule (IPL), anterior midcingulate cortex, medial orbitofrontal cortex (MOFC), and anterior insula (AI), along with networks like the left frontoparietal network (L-FPN) and midcingulo-insular network (M-CIN) [79,80,81,82,83]. Negative symptoms are associated with reduced brain volume in the prefrontal cortex, including the orbitofrontal, medial, and lateral cortices, as well as the limbic and parietal cortices [84]. Functional abnormalities involve disrupted communication, dysfunctional networks, hypometabolism, reduced activation, and hypoperfusion in various brain regions [84]. These findings highlight significant overlaps between CF-related structures and negative symptoms, emphasizing the interconnected nature of brain structures in cognitive and symptomatic domains, with consistent involvement of DLPFC, MOFC, right superior, and inferior frontal gyri in both patients with deficit syndrome (DS) and CF [83,84,85]. Increased connectivity in DS patients’ right putamen regions, along with regional cerebral blood flow patterns in the right middle frontal cortex and right inferior parietal cortex, further underscores the convergence of structural and functional aspects [84]. These shared brain regions highlight potential interconnected mechanisms and might underscore a shared physiological foundation between cognition and negative symptoms.

Our findings have important clinical implications. Negative symptoms significantly impact psychosocial functioning [5,70,86]; thus, since deficits in executive functions and particularly in cognitive flexibility affect these symptoms, we could anticipate that interventions aimed at improving cognitive deficits have a positive impact on negative symptoms as well. Reducing negative symptomatology in psychosis is expected to enhance patients’ functioning, which remains a major unresolved challenge in the treatment of psychosis today.

There are numerous cognitive remediation (CR) programs with a specific focus on enhancing executive functions. It is evident that improvements in executive functions through CR are closely linked to enhancements in real-world daily living skills and social functioning [87,88]. This relationship is supported by findings that demonstrate executive functions as a critical target to improve social functioning in psychosis. For instance, deficits in executive functions have been significantly associated with impairments in general quality of life and health-related quality of life in chronic schizophrenia patients [89]. According to our findings, we can assume that negative symptoms mediate, at least partially, the impact of executive dysfunction on functioning in FEP patients. On that score, improvement in negative symptoms may constitute an important marker of patients’ improvement in CR interventions in FEP. Therefore, testing of the efficacy of CR interventions should include the assessment of negative symptoms.

This is aligned with the growing body of literature that questions the traditional focus on positive symptoms in defining psychosis. In the study by Engen et al., the groups with no (NNS) or severe negative symptoms (SNS) demonstrated distinct trajectories in global functioning and cognitive abilities. The observation that the NNS group showed cognitive functioning comparable to healthy controls, while the SNS group experienced more pronounced cognitive deficits, is particularly telling. It underscores the intrinsic link between negative symptoms and cognitive impairments in psychosis. It highlights the need for further exploration into how interventions targeting cognitive deficits could potentially alleviate negative symptoms, thereby improving overall functioning in individuals with psychosis. Moreover, these findings support the notion that negative and cognitive symptoms may be more specific and relevant to psychosis than positive symptoms, since the functional outcomes in patients with first-episode psychosis (FEP) ten years after the onset of the illness were determined by the interplay between cognitive deficits and negative symptoms, aligning with the original [70].

While our study provides valuable insights, we should acknowledge certain limitations. The data utilized in our analysis were obtained from the ‘Athens First-Episode Psychosis Research Study’ conducted between 2015 and 2020. However, the retrospective study design and the use of pre-existing data introduce constraints, such as the potential for incomplete records or the inability to explore variables not originally considered in the prior study. The retrospective nature also poses challenges in capturing real-time fluctuations and considering changes in diagnostic criteria or treatment advances over time. It is noteworthy though that, according to our knowledge, there have not been any groundbreaking changes in these domains since the study period. The modest size of our final sample constrained our ability to include an extensive array of predictors in our analyses. The univariate regression analysis, while necessary for variable selection, may have excluded potential predictors that could have demonstrated significance in a more comprehensive multiple regression model. To mitigate this possibility, a lenient threshold of *p* < 0.2 was employed. A larger sample size would have allowed for a more exhaustive exploration of potential predictors, providing a more complete understanding of the factors influencing negative symptomatology in the context of cognitive deficits.

Future research could explore the mechanisms underlying the relationship between executive functions and negative symptoms, employing longitudinal studies with larger, diverse samples to enhance the generalizability of the findings. This continued exploration will contribute to a more comprehensive understanding of the dynamic interplay between cognitive deficits and symptom outcomes in psychosis. Our study advocates for more theory-driven, in-depth research into negative and cognitive symptoms and their neuroscientific underpinnings. This includes exploring their associations with functional outcomes and potential biomarkers, including genetic and brain imaging data.

## 5. Conclusions

Our results emphasize the importance of cognitive deficits, and in particular cognitive flexibility as a key component of executive functions, in predicting the development and progression of negative symptoms in individuals with FEP. By identifying specific cognitive markers in the early stages of psychosis, clinicians and researchers can pave the way for targeted interventions oriented towards improving functional outcomes and enhancing the quality of life for individuals navigating the challenging path of psychotic illness.

## Figures and Tables

**Table 1:** Demographic, clinical characteristics, and duration of untreated psychosis, according to gender in the sample.

Characteristics		Women (*n* = 26)			Men (*n* = 37)		*p*-Value
	Mean	SD	Range	Mean	SD	Range	
Age	27.92	9.33	17–46	25.16	7.00	16–46	0.208
DUP	26.77	52.91	0–260	34.35	73.00	2–384	0.634
PANSS general psychopathology	28.69	9.60	17–66	30.60	9.65	18–58	0.443
PANSS positive	13.38	5.56	7–35	13.89	4.59	7–25	0.704
PANSS negative	12.50	6.48	7–34	15.43	5.90	7–27	0.072
Chlorpromazine Equivalent	312.50	176.95	0–800	295.27	251.03	0–1250	0.750

DUP = duration of untreated psychosis, PANSS = positive and negative syndrome scale, SD = standard deviation.

**Table 2:** Simple regression results between each variable and the 1-year PANSS negative score * *p* < 0.2, ** *p* < 0.05.

Variable	β	SE	*t*-Value	*p*-Value
1-month PANSS negative	0.4201	0.0958	4.3835	3.65073 × 10^−5^ **
DUP	0.0375	0.0104	3.6127	0.0005 **
1-month PANSS positive	0.3382	0.1305	2.5918	0.0114 **
Categories	−0.921	0.3625	−2.5399	0.0133 **
Age	0.1824	0.0775	2.3525	0.0212 **
Chlorpromazine equivalents	0.0063	0.0027	2.2876	0.0249 **
Academic PAS	6.6286	3.2776	2.0224	0.0469 **
Percent conceptual level response	−0.0709	0.0351	−2.0173	0.0474 **
Percent errors	0.0892	0.0457	1.9514	0.0550 *
Gender	−2.4388	1.2971	−1.8802	0.0639 *
1-month PANSS general psychopathology	0.1286	0.0694	1.8530	0.0677 *
VCI	−0.0858	0.0521	−1.6459	0.1039 *
Premorbid IQ	−0.3173	0.2161	−1.4683	0.1462 *
Perseverative errors	0.0650	0.0460	1.4120	0.1623 *
Percent perseverative responses	0.0629	0.0490	1.2850	0.2030
Percent perseverative errors	0.0729	0.0641	1.1378	0.2590
FSIQ	−0.0439	0.0522	−0.8411	0.4029
Social PAS	2.8177	3.5341	0.7973	0.4279
TMT A	0.0313	0.0410	0.7632	0.4478
WMI	−0.0313	0.0479	−0.6535	0.5154
PRI	−0.0180	0.0454	−0.3972	0.6923
PSI	0.0154	0.0466	0.3314	0.7412
Education years	0.0374	0.3211	0.1164	0.9077
TMT B	0.0008	0.0135	0.0647	0.9486

Variables are listed according to the significance of their association. SE = standard error, PANSS = positive and negative syndrome scale, DUP = duration of untreated psychosis, categories = categories completed in WCST, PAS = premorbid adjustment scale, VCI = verbal comprehension index, FSIQ = full-scale IQ, TMT = trail making test, WMI = working memory index, PRI = perceptual reasoning index, PSI = processing speed index, * *p* < 0.2, ** *p* < 0.05.

**Table 3:** Final multiple regression model for prediction of negative symptoms at 1-year follow-up. Final model: adjusted R^2^ = 0.38, F = 13.62, *p* < 0.001.

Variables	β	*t*	*p*	95% CI	
				Lower Bound	Upper Bound
DUP	0.032	3.608	0.0006 **	0.015	0.049
1-month PANSS negative	0.344	3.791	0.0004 **	0.162	0.526
Categories	−0.812	−2.646	0.0104 *	−1.414	−0.212

β = beta coefficient or partial regression coefficient, *t* = *t*-value, *p* = *p*-value, CI = confidence interval, DUP = duration of untreated psychosis, PANSS = positive and negative syndrome scale, categories = categories completed in WCST, * *p* < 0.05, ** *p* < 0.01.

## Data Availability

Data are available upon request. The data are not publicly available due to privacy concerns related to participant information.
